# Dissecting Cardiovascular Responses to a Fixed‐Interval Volitional Sighing Protocol Using a Mixed Modeling Approach

**DOI:** 10.1111/psyp.70235

**Published:** 2026-01-16

**Authors:** Neel Muzumdar, Kelly Sun, Samuel Zhang, Kelsey Piersol, Anthony P. Pawlak, Marsha E. Bates, Jennifer F. Buckman

**Affiliations:** ^1^ Department of Kinesiology and Health Rutgers University—New Brunswick New Brunswick New Jersey USA

**Keywords:** blood pressure, breathwork, heart rate variability, pulse, respiration, sighing

## Abstract

Sighing generates a reliable sympathetic cardiovascular response that, like exercise, could be leveraged in a graded “stress test” to reveal preclinical changes in cardiovascular health and stress reactivity. This study presents the fixed‐interval volitional sighing (FIVS) protocol, which rhythmically paces sighs at different frequencies to systematically load the cardiovascular system. Cardiovascular and autonomic responses during the FIVS protocol were statistically dissected to independently characterize physiological responses. Sex differences were explored as a preliminary step toward characterizing factors that affect sigh reactivity. Healthy college students (*n* = 250, 65% female) completed a baseline task and two sighing tasks: a longer inter‐sigh interval task (1 sigh per 30 s, long interval), followed by a shorter inter‐sigh interval task (1 sigh per 15 s, short interval). Heart rate (HR), blood pressure, and respiration were continuously measured. Mixed models with a priori cardiorespiratory assumptions isolated HR, low‐frequency heart rate variability (LF‐HRV), high‐frequency HRV (HF‐HRV), pulse transit time variability (PTTv), mean arterial pressure (MAP), low‐frequency blood pressure variability (LF‐BPV), and high‐frequency BPV (HF‐BPV) responses to the sighing tasks. HR, LF‐HRV, PTTv, MAP, and LF‐BPV increased significantly from baseline to both sighing tasks, with greater changes observed during short‐interval sighing. HF‐BPV increased similarly from baseline to both sighing tasks. HF‐HRV decreased only during the short‐interval sighing task. Males exhibited greater increases than females in HR, LF‐HRV, LF‐BPV, HF‐BPV, and PTTv but smaller decreases in HF‐HRV in response to sighing. Volitional sighing elicits cardiac, vascular, and autonomic responses consistent with sympathetic activation. As time under load and loading intensity increased, greater responses were observed in several vascular and sympathetic indices. Sex differences suggest that the FIVS protocol can detect person‐specific differences in cardiovascular responding. Sighing is physically accessible for most people, and the FIVS protocol may be useful as a stress test to detect early‐stage cardiovascular or autonomic dysfunction.

## Impact Statement

Sighing produces a robust physiological response that could serve as a physically accessible method for cardiovascular assessment. This study introduces a volitional sighing protocol as a graded “stress test,” wherein rhythmically paced sighs reliably activate sympathetic cardiovascular responses in a dose‐dependent manner. These findings suggest that this challenge paradigm can visibly and reliably provoke cardiac, vascular, and autonomic responses and do so differentially in males and females.

## 1 Introduction

Sighing is a common yet understudied respiratory behavior that is observed throughout the lifespan. It is a distinct respiratory phenomenon, with separate neural control centers (Cui et al. 2025; Ashhad et al. 2022), different physiological response patterns (Vaschillo et al. 2015), and complex psychological concomitants (Vlemincx et al. 2022). A naturalistic sigh can initiate arousal, and sighing becomes more frequent when arousal is high (Vlemincx et al. 2013). Thus, a sigh can serve as a stimulus for arousal and a response to arousal. In both cases, the sigh is coupled with an acute sympathetic cardiovascular response (Severs et al. 2022), followed by a temporary sense of relief or relaxation (Vlemincx et al. 2016).

The main theory about the psychophysiological value of sighing proposes that a naturalistic sigh acts as a “resetter,” re‐equilibrating physiological systems that have become erratic (Vlemincx et al. 2013). The sharp, deep inhalation during a sigh causes an abrupt acceleration in heart rate (HR), which increases blood flow and consequently elevates mean arterial pressure (MAP) (Chaudhry et al. 2025). Such cardiovascular mobilization is indicative of a sympathetic response. This bolus of sympathetic activity, however, ultimately serves to rebalance autonomic function toward a more restful (parasympathetic) state (Vlemincx et al. 2016, 2022), and a return to physiological stability is psychologically experienced as relief and relaxation (Critchley and Harrison 2013). This idea aligns with allostatic theory (Sterling 2012), which posits that phenomena that keep the working range of a system broad are health promoting by ensuring greater system capacity to respond to challenges.

Sighing parallels the effects of exercise on cardiorespiratory function, wherein acute sympathetic activation during exercise can promote postexercise emotional (Giles et al. 2018) and autonomic (Fu and Levine 2013) regulation. Also like exercise, sighing can elicit arousal (Ramirez 2014) as well as dampen arousal elicited by extrinsic factors (e.g., stress, negative affect). In cardiology, aerobic exercise is used as a “stress test,” wherein the cardiovascular system is loaded with a progressive exercise protocol to unmask health issues that are not detectable when the system is at rest. We propose that sighing protocols could likewise be developed to provoke sympathetic responses that could uncover latent physiological changes that are not yet clinically evident (Vaschillo and Vaschillo 2020). Sighing is physically accessible to nearly everyone, regardless of health or disability status, and requires only standard cardiovascular equipment (e.g., wearables and clinical‐grade electrocardiography [ECG]). Building on preliminary data (Vaschillo et al. 2015, 2018; Vaschillo and Vaschillo 2020), this study sought to characterize a novel graded sighing protocol as a preliminary step toward evaluating its feasibility as a “stress test” to identify preclinical changes in cardiovascular health and stress reactivity associated with unhealthy but common lifestyle factors, like substance use and poor sleep.

### 1.1 Fixed‐Interval Sighing to Provoke Stress Responses

The naturalistic sigh has proven difficult to fully reproduce (Vlemincx et al. 2010) and its inherent infrequency makes it difficult to study. Thus, various breathing techniques that differ in depth, pattern, and frequency have been developed to deliberately emulate the putative health benefits of sighing. For example, an online study using wrist‐worn wearable technology reported that 1 month of daily cyclic sighing was associated with reduced anxiety and improved parasympathetically‐mediated heart rate variability (HRV) (Balban et al. 2023), a cardiovascular indicator of physical and psychological well‐being (Shaffer et al. 2014). However, the cyclic breathing instructions did not perfectly parallel known respiratory mechanisms of sighing (e.g., double nasal inhale), and only subacute changes were evaluated. Moreover, that sighing protocol was designed as an intervention, not an experimental stress protocol.

This study employed a graded fixed‐interval volitional sighing (FIVS) protocol wherein each consciously produced sigh involved a deep and sharp inhale followed by a natural exhale. Prior work demonstrated that these instructions led to sigh‐like behaviors that elicited an acute change in HR (during inhalation) and distal blood pressure (postinhalation) similar to that of a spontaneous sigh (Vaschillo et al. 2015). However, unlike naturalistic sighing, which is paced on a minutes‐scale (Posada‐Quintero et al. 2016; Task Force of The European Society of Cardiology and The North American Society of Pacing 1996) and typically occurs in the backdrop of heightened sympathetic tone, the FIVS protocol demands substantially more frequent sighs and does so in the absence of external stressors (i.e., it acts as a stimulus, not a response). Preliminary evidence suggested that repetitive sigh generation during short‐interval FIVS protocols can challenge the cardiovascular system, producing high interindividual variability that may serve as a marker of cardiovascular health (Vaschillo and Vaschillo 2020; Vaschillo et al. 2018).

Based on allostatic load theory (McEwen 1998) and exercise‐based stress testing protocols, we created an experimental FIVS protocol to build allostatic pressure on the cardiovascular system over time and with predetermined intensity jumps. The design intentionally progresses from an initial lower‐intensity 5‐min period wherein sighs were spaced 30 s apart interspersed with eupneic breathing (long‐interval sighing) to a higher‐intensity 5‐min period with sighs every 15 s interspersed with eupneic breathing (short‐interval sighing). This parallels common stress tasks that start with a “warm up,” or lower‐demand task, and then build intensity over time. To adhere to cardiovascular psychophysiology measurement and short‐term analysis standards, 5‐min intervals were used. Since each sigh generates a sympathetic response followed by passive recovery (Vaschillo et al. 2015) and this cycle appears to be nonhabituating, we hypothesized that the short‐interval sighing task would elicit greater cardiovascular and autonomic responses compared to the high‐interval sighing task, due to both the time under load and/or the intensification of the task.

### 1.2 Exploring Sex Differences in Stress Responses

Preliminary evidence suggested that faster sighing can sometimes overload the cardiovascular system, leading to a loss of sympathovagal balance rather than a reset (Vaschillo and Vaschillo 2020). This system overload was proposed to be an early indicator of vascular deterioration (Vaschillo and Vaschillo 2020). The present study extends these observations by comparing cardiovascular and sympathetic responses in ostensibly healthy male and female young adults based on sex differences in cardioprotective mechanisms to acute arousal observed in both human and animal studies. Specifically, females typically demonstrate higher baseline heart rate (i.e., sympathetic tone) and greater HRV in the high frequency range (HF‐HRV; parasympathetic tone) than males at baseline, but, under stress, exhibit smaller sympathetic responses (Koenig and Thayer 2016; Carnevali et al. 2023). Exploring how healthy males and females differently respond to sighing, which elicits sympathetically driven cardiovascular responses, can serve as a foundation for studying other individual differences factors and behavioral health factors that may serve to drive stress responding. We hypothesized that females would exhibit smaller changes in cardiovascular activity during the FIVS protocol than males.

### 1.3 Deconstructing Cardiovascular Indices to Characterize Stress Responding and Recovery

The breadth of subjective and objective effects of sighing, along with the magnitude of the physiological “reset” it triggers, suggests broad cardiac, vascular, and autonomic involvement that, along with factors such as blood flow, enable the cardiovascular system to dynamically maintain homeostasis. However, it is difficult to isolate control processes with noninvasive measurement tools because they are fundamentally interwoven and redundant in a state‐dependent manner. Thus, this paper advances prior research by using a mixed modeling approach to dissect the cardiovascular responses to sighing with a priori cardiorespiratory assumptions and covariates.

From the ECG, heart rate (HR) provides a snapshot of metabolic demand driven by the ratio of sympathetic and parasympathetic activity as the heart is under the influence of both branches of the autonomic nervous system. HRV further provides insight into the heart's dynamic response capacities by characterizing natural fluctuations in the heart rhythm that are driven, beat by beat, by internal and external stimuli. Importantly, the HRV signal can be decomposed into frequency bands that better map onto autonomic function. Changes in the high frequency band of the R‐to‐R interval spectrum (HF‐HRV), for example, are driven by parasympathetic (vagal) modulation of cardiac functioning (Task Force of The European Society of Cardiology and The North American Society of Pacing 1996). Respiratory sinus arrhythmia (RSA) (Garcia et al. 2013), i.e., the synchronization of cardiac variability to respiration, is a vagal mechanism that predominates the HF‐HRV signal during normal respiration. Conversely, the low‐frequency band of the R‐to‐R interval spectrum (LF‐HRV) approximates other cardiovascular control processes; during regular breathing, baroreflex activity predominates this signal, but during controlled breathing (depending on breathing rate) either sympathetic and/or parasympathetic activity can influence the signal (Vaschillo et al. 2015; Shaffer et al. 2014).

Pulse transit time (PTT), or the travel time of blood between two pulse sites, is a gold standard noninvasive vascular function assessment and predominantly reflects sympathetic function (Gordan et al. 2015). Mean PTT and PWV (a PTT derivative for which distance is controlled) reflect static vascular tone, whereas PTT variability (PTTv) provides a window into short‐term vasomotor responses (An et al. 2021). Likewise, systolic blood pressure variability (BPV), also derived from the vascular signal, offers insight into vascular dynamics. In particular, the LF band (LF‐BPV) is associated with sympathetic vascular control (Parati et al. 1995); the HF band of BPV (HF‐BPV) is likely influenced by respiratory mechanisms such as RSA and tidal volume (Parati et al. 1995; deBoer et al. 1987).

This study hypothesized that the independent contributions of cardiac and vascular responses as well as both sympathetic and parasympathetic roles in sighing could be isolated using statistical strategies. For example, RSA, which was proxied as the time delay between peak respiration and peak R–R interval, i.e., when heart rate is slowest, was added as a constant covariate to appropriate models of sighing responses. To isolate the effect of sighing on cardiac versus vascular function, models of HR controlled for RSA and PWV. To more directly attribute the effects of sighing on HRV to autonomic functioning (Yasuma and Hayano 2004), HF‐HRV models controlled for RSA and LF‐HRV, while LF‐HRV models controlled for HF‐HRV and RSA. These covariates control respiratory‐driven shifts in power from the HF to LF band that occur with decreased respiration rate, ensuring that observed changes reflect autonomic, rather than respiratory, influences. To better characterize how sighing affects vascular dynamics, models of PTTv controlled for HR. Changes in HR are closely associated with changes in PTTv (Zhang et al. 2015; Drinnan et al. 2001), likely due to the fact that the vasculature needs to adapt to blood flow changes driven by cardiac activity. To assess the effects of sighing on blood flow, the effects of HR and PWV were controlled in models of MAP because, in the absence of HR or vascular influences, the largest influence on MAP is stroke volume (DeMers and Wachs 2025). To parallel analyses of HRV, LF‐BPV models controlled for RSA and HF‐BPV; HF‐BPV models controlled for RSA and LF‐BPV. Finally, together, these models statistically parse the effects of FIVS on HR, LF‐HRV, HF‐HRV, PTTv, MAP, LF‐BPV, and HF‐BPV to holistically evaluate stress responding to repetitive sighing.

## 2 Methods

### 2.1 Participants

Physically healthy, normotensive undergraduate students (*n* = 250) with at least 2 years until graduation were recruited into an ongoing 2‐year prospective study of how college lifestyle behaviors (e.g., drinking, exercise, sleep) affect cardiovascular health. Participants (65% female) were 17–21 years old. Individuals were excluded if they self‐reported medical conditions that affected cardiovascular functioning or were prescribed medications known to affect cardiovascular tone. Individuals with self‐reported psychotic disorders (e.g., schizophrenia) or a body mass index (BMI) > 35 or < 16 were also excluded. A survey battery and a cardiovascular assessment at the start and end of the 2‐year study period bookended brief weekly online surveys about lifestyle behaviors and subjective wellness. The present study uses cardiovascular data from one cardiovascular assessment; weekly behavioral data collection is ongoing. Recruitment was conducted through distribution of flyers, online posts, and word of mouth. This study was approved by the Rutgers University ArtSci Institutional Review Board, Study ID: Pro2018001823.

### 2.2 Design

Following written consent, laboratory sessions began with a baseline survey (e.g., demographics) and physical measurements (e.g., height, weight, arm length). A standard lead II ECG configuration was used to collect data via a PowerLab Acquisition System (ADInstruments, Colorado Springs, CO). A cuff sensor for beat‐to‐beat blood pressure measurement was attached to the second phalange of the right middle finger; the data were collected using a Finometer MIDI (Finapres Medical Systems, Enschede, Netherlands). A stretch belt with piezo‐sensor for collection of respiratory data was set around the chest. Collected data were sampled at 2000 Hz. During recordings, the participants were seated upright in a quiet environment at room temperature. Participants were instructed to rest sufficiently and maintain their regular diet before the laboratory session. They were asked avoid substance use within 24 h and caffeine use within 2 h of the experimental session. Laboratory sessions were available on weekdays from 9 a.m. to 5 p.m.; 98% occurred between 10 a.m. and 3:30 p.m.

Physiological data were collected during four 5‐min experimental tasks. Data from the first three tasks: a baseline task (BL), a long‐interval sighing task, and a short‐interval sighing task, were analyzed for this study. The 5‐min analysis segments, with 1‐min inter‐task periods, were selected because they provide sufficient duration for reliable measurement of low‐frequency oscillations while maintaining participant engagement and minimizing fatigue‐related confounds. Given that the protocol aimed to assess cardiovascular responses under progressively higher demands, complete physiological recovery between tasks was not required and individual differences in recovery were expected. Further, for this reason, the tasks were not randomized; this parallels standard cardiovascular assessment tools (e.g., cardiology stress testing and human performance testing). A brief training session occurred prior to the breathing tasks to ensure reliable performance and minimize psychological distress that may be associated with anticipation of a challenging task. Sigh training concluded when participants successfully performed a volitional sigh at least twice consecutively and verbally expressed that they were ready to begin.

A low cognitive demand baseline task (Jennings et al. 1992) was administered first. A rectangle was presented on the television screen and changed color every 10 s. Participants were instructed to count the number of blue rectangles, with the recommendation to be liberal with shading (e.g., blue versus blue–green) and hue (e.g., light versus dark blue). Two sighing tasks followed, wherein participants inhaled quickly and deeply through their mouth when cued by a large red square and then exhale before the red square vanished (2 s). The first sighing task (long‐interval sighing) involved a volitional sigh every 30 s; this was the lower‐intensity task. The second, higher‐intensity sighing task involved a volitional sigh twice as often: every 15 s (short‐interval sighing). Naturally, sighing occurs approximately every 5 min. Normal respiration, which was visually confirmed, occurred between each volitional sigh.

### 2.3 Data Postprocessing

Following a low pass filter at 10 Hz, all signals underwent manual artifact correction to remove nonphysiological ectopic beats and lost spikes due to movement artifacts. R–R Intervals (RRI) were calculated as the time difference (in milliseconds) between two ECG R‐wave peaks. A proxy for RSA, i.e., cardiorespiratory synchronization, was calculated as the time difference (in seconds) between the peak of the respiration and the subsequent RRI signal peak (HR trough). Longer time delays between peak respiration and RRI suggest stronger RSA, i.e., more synchronization, as this translates to shorter time delays between peak inhalation and heart rate. RSA was calculated for every breath in the baseline task, but only for each sigh during each sighing task.

Mean arterial pressure was extracted directly from the continuous arterial pressure signal. PTT was calculated as the time difference (in milliseconds) between the peak of the ECG R‐wave and the subsequent raw BP signal peak (Ding and Zhang 2019). To create the PTT time series data, each PTT data point was aligned with its corresponding BP peak. Data points within BP calibration periods were excluded from analysis. PWV was calculated as arm length (difference between pulse measurement sites) divided by PTT.

### 2.4 Spectral Density Calculation

Fast Fourier transformations were applied to the RRI and BP time series using ANSLab (Blechert et al. 2016) after resampling to 4 Hz, and power spectral density graphs were created to obtain the distribution of power across distinct frequencies. Spectral analysis was performed using Welch's method with a 60‐s window size and 50% overlap (30 s). An FFT size of 2048 points was applied with zero‐padding as necessary. Per standard procedure (Task Force of The European Society of Cardiology and The North American Society of Pacing 1996), spectral data underwent a natural logarithmic transformation to satisfy assumptions for parametric statistical analyses. LF‐HRV and LF‐BPV were designated at 0.04–0.15 Hz, and HF‐HRV and HF‐BPV were designated at 0.15–0.4 Hz. While 5‐min intervals is considered the gold standard for short‐term analysis of LF‐HRV (Task Force of The European Society of Cardiology and The North American Society of Pacing 1996), they are too short for reliable evaluation of very‐low‐frequency HRV (VLF‐HRV); therefore, this band was excluded from analysis from both HRV and BPV analyses.

### 2.5 Statistical Analysis

Linear mixed models were conducted using SAS 9.4 PROC MIXED (SAS Institute, Cary, NC) to evaluate within‐person physiological changes from baseline (reference task) to the sighing tasks (long‐interval, then short‐interval) and between‐group sex differences. The model for HR controlled for PWV to account for vascular‐driven effects and for RSA to account for respiratory effects. The model for HF‐HRV controlled for RSA and LF‐HRV, and the model for LF‐HRV controlled for RSA and HF‐HRV, as slower breathing rates can shift power in the RRI spectrum. The model for PTTv controlled for HR. The model for MAP controlled for PWV and HR to estimate changes blood flow (driven primarily by stroke volume) in isolation. The models for BPV mirrored HRV (e.g., HF‐BPV controlled for RSA and LF‐BPV).

Interactions between task and sex were used to evaluate between‐group differences, and controlled variables were included as main effects. Pairwise comparisons were conducted regardless of model significance to better evaluate graded responses to increased sigh frequency. Box plots were generated and accompanied by jittered scatter plots of the data using SAS 9.4 PROC SGPLOT to depict significant changes across tasks. Influential data points were visually assessed and removed if Cook's *D* > 1, RLD > 1.5, studentized residuals > |4|, or doing so improved the Q–Q plots for each model. Further details of influence analysis, as well as initial models with versus without covariates, are included in supplemental materials. All statistical analyses were conducted to determine cardiovascular and autonomic changes by volitional sighing.

## 3 Results

**FIGURE 1:**
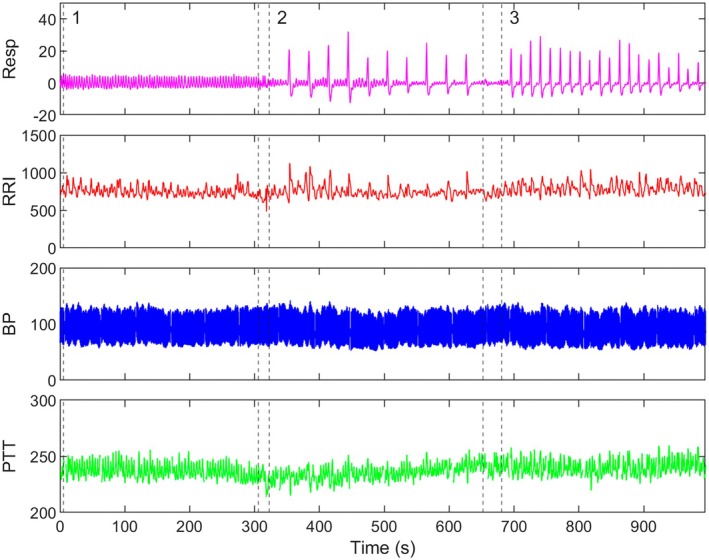
Sample physiological data collection during the laboratory session. Respiration (pink) and BP (blue) were collected during the laboratory session; RRI (red) and PTT (green) were calculated postsession. Tasks were performed in a fixed order: Baseline (1), long‐interval FIVS (2), and short‐interval FIVS (3).

This sample included 250 college students (65% female), aged 19 ± 1 years. Table 1 includes average baseline cardiovascular measures. Figure 1 shows representative data from one participant across baseline, long‐interval sighing task, and short‐interval sighing task, in that order. Volitional sighs appeared as respiratory traces (top panel) similar to naturalistic sighs; cardiac (RRI) and vascular (BP, PTT) responses to the FIVS tasks are observable as changes in mean or variability.

**TABLE 1:** Average baseline cardiovascular measures.

Cardiovascular measure	Mean ± SD (male)	Mean ± SD (female)
HR (bpm)*	69.68 ± 9.16	76.36 ± 10.52
LF‐HRV (log)*	9.32 ± 0.85	8.80 ± 0.83
HF‐HRV (log)	8.19 ± 1.03	8.14 ± 1.08
MAP (mmHg)	83.56 ± 12.53	80.37 ± 14.77
PTTv (ms)*	36.23 ± 18.51	30.78 ± 16.50

* Significant (*p* < 0.05) baseline sex differences from *t*‐tests.

### 3.1 Response to an Individual Sigh

**FIGURE 2:**
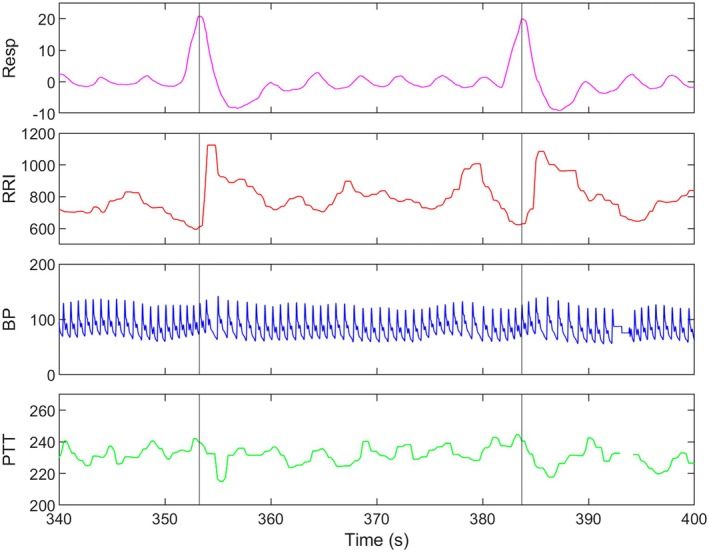
Sample physiological data during the long‐interval FIVS task that is representative of the modal response pattern during a volitional sigh. Peak inhalation (pink) during a volitional sigh aligned closely with the RRI (red) trough, PTT (green) peak, followed by a delayed BP (blue) increase.

As expected, individual volitional sighs resulted in phasic HR, BP, and PTT responses with distinct time courses (Figure 2). Specifically, as inhalation starts, HR begins accelerating (i.e., decreasing R‐to‐R intervals) concomitantly with vessel dilation (i.e., increasing PTT) and BP increases. Maximal HR (i.e., shortest RRI) was observed in phase with the peak inhalation during a volitional sigh. Maximal vasodilation was also in phase with or immediately preceding peak inspiration volume. The rise in BP peaked approximately 2–3 beats after peak inhalation in parallel to a sharp drop in HR and vasoconstriction (i.e., shortening PTT). Figure 2 shows two sighs from the long‐interval sighing task; with sighs paced every 30 s, there was ample recovery time and numerous natural breaths between each sigh.

**FIGURE 3:**
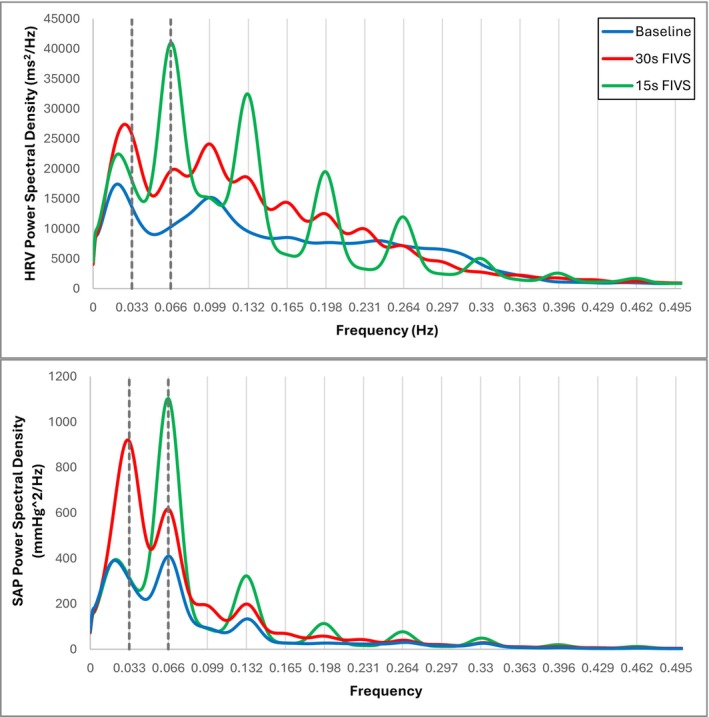
Group‐averaged heart rate variability (HRV, top) and systolic arterial pressure (SAP, bottom) frequency spectra calculated during baseline (blue), long‐interval FIVS (30s interval, red), and short‐interval FIVS (15s interval, green). Vertical dashed lines distinguish 0.033 and 0.066 Hz frequencies.

Figure 3 presents group‐averaged HRV spectra from each sighing task to show that rhythmical sighing at a specific pace elicited a reliable cardiovascular response at the same frequency (i.e., sighing every 30 s elicited an oscillation in RRI with a period of 30 s). There was also evidence of an attenuating sinusoidal oscillatory pattern at multiples of the sighing frequency, suggestive of harmonics.

### 3.2 Response to the FIVS Tasks

A series of mixed models found significant effects of sighing on cardiovascular functioning. Table 2 includes a summary of model results.

**TABLE 2:** Summary of main effects of task and sex, as well as the task × sex interaction.

Cardiovascular measure	Response to sighing tasks	Overall sex differences	Task × sex interaction
HR	BL < long < short	M < F	F = less change
LF‐HRV	BL < long < short	F < M	F = less change
HF‐HRV	BL = long > short	—	F = more change
PTTv	BL < long < short	F < M	F = less change
MAP	BL = long < short	—	—
LF‐BPV	BL < long < short	F < M	F = less change
HF‐BPV	BL < long = short	F < M	F = less change

Abbreviations: BL, baseline; F, female; M, male; long, long (30 s) interval sighing task; short, short (15 s) interval sighing task.

**FIGURE 4:**
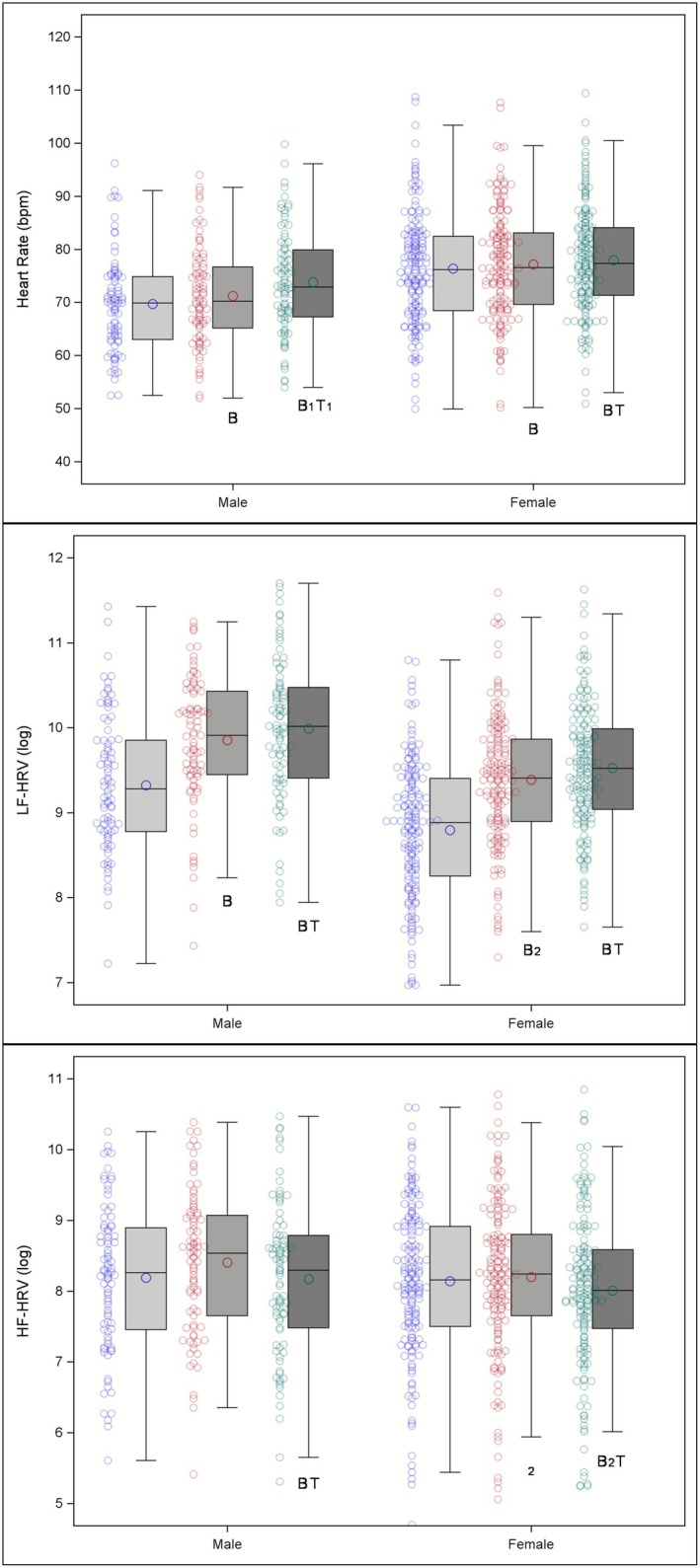
Graphical depiction of cardiac responses during baseline (light gray), long‐interval FIVS (mid‐gray), and short‐interval FIVS (dark gray) in males and females; individual‐level data shown to the left of each bar. When controlling for a priori selected covariates, significant differences between FIVS tasks and baseline are denoted by a letter “B.” Significant differences between FIVS tasks are denoted by a letter “T.” Numerical subscripts denote significant sex × task interactions. Letters with a subscript “1” indicate between‐task changes that were greater in males than in females. Letters with a subscript “2” indicate between‐task changes that were greater in females than in males.

In mixed models designed to isolate the effects of sighing on HR (controlling for PWV and RSA), there was a main effect of task on HR, *F* (2,451) = 54.15, *p* < 0.0001. Pairwise comparisons (Figure 4) showed that HR was lower at baseline compared to the long‐interval sighing task, *t* (451) = 4.25, *p* < 0.0001, and the short‐interval sighing task, *t* (451) = 7.78, *p* < 0.0001. HR was also lower during the long‐ versus short‐interval sighing tasks, *t* (451) = 8.74, *p* < 0.0001. Exploratory comparisons of sex differences revealed that females had faster HR than males, *F* (1,451) = 20.46, *p* < 0.0001. A significant sex × task interaction, *F* (2,451) = 10.02, *p* < 0.0001, suggested that females showed less increase in HR than males from BL to the short‐interval sighing task, *t* (451) = −3.82, *p* = 0.0005, from the long‐ to short‐interval sighing tasks, *t* (451) = −4.20, *p* < 0.0001. There was no significant sex difference for the change between BL and the long‐interval sighing task.

In mixed models designed to isolate the effects of sighing on LF‐HRV (controlling for RSA and HF‐HRV), there was a main effect of task on LF‐HRV, *F* (2,465) = 119.70, *p* < 0.0001. Pairwise comparisons (Figure 4) showed that LF‐HRV was significantly lower at baseline compared to the long‐interval sighing task, *t* (465) = 5.84, *p* < 0.0001, and the short‐interval sighing task, *t* (465) = 8.74, *p* < 0.0001. LF‐HRV was also significantly lower during the long‐ versus short‐interval sighing task, *t* (465) = 5.48, *p* < 0.0001. The sex difference analysis showed that females had lower LF‐HRV than males, *F* (1,465) = 48.18, *p* < 0.0001. The overall sex × task interaction was not significant, but LF‐HRV increased more from baseline to the long‐interval sighing task for females than for males, *t* (465) = 2.00, *p* = 0.046.

In mixed models designed to isolate the effects of sighing on HF‐HRV (controlling for RSA and LF‐HRV), there was a main effect of task on HF‐HRV, *F* (2,463) = 71.53, *p* < 0.0001. Pairwise comparisons indicated that HF‐HRV was lower during the short‐interval sighing task compared to baseline, *t* (463) = −5.46, *p* < 0.0001, and the long‐interval sighing task, *t* (463) = −7.28, *p* < 0.0001. The main effect of sex on HF‐HRV was not significant (Figure 4), but there was a significant sex × task interaction, *F* (2,463) = 3.44, *p* = 0.033, showing that HF‐HRV decreased more (from baseline) for females than males during both the long‐interval, *t* (463) = −2.62, *p* = 0.009, and short‐interval sighing task, *t* (463) = −1.97, *p* = 0.049. There was no significant sex difference for HF‐HRV change between the long‐ and short‐interval sighing tasks.

**FIGURE 5:**
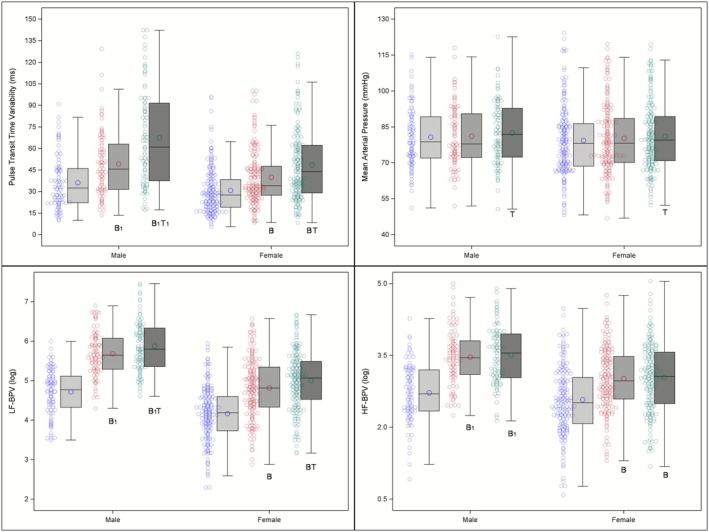
Graphical depiction of vascular responses during baseline (light gray), long‐interval FIVS (mid‐gray), and short‐interval FIVS (dark gray) in males and females; individual‐level data shown to the left of each bar. When controlling for a priori selected covariates, significant differences between FIVS tasks and baseline are denoted by a letter “B.” Significant differences between FIVS tasks are denoted by a letter “T.” Numerical subscripts denote significant sex × task interactions. Letters with a subscript “1” indicate between‐task changes that were greater in males than in females. Letters with a subscript “2” indicate between‐task changes that were greater in females than in males.

In mixed models designed to isolate the effects of sighing on PTTv (controlling for HR), there was a main effect of task on PTTv, *F* (2,456) = 98.96, *p* < 0.0001. PTTv was lower at baseline compared to the long‐interval sighing task, *t* (456) = 7.05, *p* < 0.0001, and the short‐interval sighing task, *t* (456) = 10.34, *p* < 0.0001. PTTv was also significantly different between the long‐ and short‐interval sighing tasks, *t* (456) = 8.39, *p* < 0.0001. The sex differences analysis (Figure 5) showed that females had lower PTTv, *F* (1,456) = 15.40, *p* = 0.0001, than males. A significant sex × task interaction, *F* (2,456) = 9.23, *p* = 0.0001, showed that PTTv changed more from baseline in males during long‐interval sighing, *t* (456) = 2.36, *p* = 0.019, and short‐interval sighing, *t* (456) = 4.19, *p* < 0.0001, FIVS tasks as well as from long‐ to short‐interval sighing, *t* (456) = 3.77, *p* = 0.0002.

In mixed models designed to isolate the effects of sighing on MAP (controlling for HR and PTTv), there was a main effect of task on MAP, *F* (2,470) = 6.69, *p* = 0.0014. Pairwise comparisons in the mixed model (Figure 5) showed that MAP was significantly higher during the short‐ versus long‐interval sighing task, *t* (470) = 2.67, *p* = 0.0078, and marginally (trending) higher versus baseline, *t* (470) = 1.79, *p* = 0.0736. MAP was not significantly different between baseline and the long‐interval sighing task. Sex differences between the FIVS tasks were not significant. The sex × task interaction was not significant.

In mixed models designed to isolate the effects of sighing on LF‐BPV (controlling for HF‐BPV and RSA), there was a main effect of task on LF‐BPV, *F* (2,460) = 112.12, *p* < 0.0001. Pairwise comparisons (Figure 5) showed that LF‐BPV was higher during long‐interval sighing, *t* (460) = 9.46, *p* < 0.0001, and during short‐interval sighing, *t* (460) = 11.31, *p* < 0.0001, than BL. The short‐interval sighing was also higher than long‐interval sighing, *t* (460) = 4.00, *p* = 0.0003. Females had lower LF‐BPV, *F* (1,460) = 68.82, *p* < 0.0001, than males. There was a significant sex × task interaction, *F* (2,460) = 3.13, *p* = 0.0445; increases in LF‐BPV were significantly smaller for females than for males when comparing changes from BL to long‐interval sighing, *t* (460) = −2.50, *p* = 0.0127, and from BL to short‐interval sighing, *t* (460) = −2.05, *p* = 0.041. There was no significant sex difference for the change between the long‐ and short‐interval sighing tasks.

In mixed models designed to isolate the effects of sighing on HF‐BPV (controlling for LF‐BPV and RSA), there was a main effect of task on HF‐BPV, *F* (2,463) = 27.94, *p* < 0.0001. Pairwise comparisons (Figure 5) showed that HF‐BPV was higher during long‐interval sighing, *t* (463) = 6.69, *p* < 0.0001, and during short‐interval sighing, *t* (463) = 5.59, *p* < 0.0001, than BL. HF‐BPV was also higher. There was no significant difference between short‐ and long‐interval sighing. Females had lower HF‐BPV, *F* (1,463) = 5.60, *p* = 0.0184, than males. There was a significant sex × task interaction, *F* (2,463) = 4.28, *p* = 0.0143; increases in HF‐BPV were smaller for females than for males when comparing changes from BL to long‐interval sighing, *t* (463) = −2.87, *p* = 0.0043, and BL to short‐interval sighing, *t* (463) = −2.66, *p* = 0.008. There was no significant sex difference for the change between long‐interval sighing and short‐interval sighing.

## 4 Discussion

This study presents the FIVS protocol that uses deliberately initiated sighs to elicit a cardiovascular sympathetic response. Our findings suggest that each sigh in the protocol generated a nonhabituating cardiovascular response that was consistent with system loading and stress responding. Further, the progressive load (5‐min of 1 sigh every 30 s followed by 5‐min of 1 sigh every 15 s) resulted in a broad pattern of small but significant cardiovascular changes from baseline that suggest that the FIVS protocol was a reliable activator of the sympathetic nervous system, even in young, ostensibly healthy adults. This task, therefore, may be a useful adjunct to current exercise‐based “stress tests” used to reveal latent abnormalities in vascular function, autonomic regulation, and hemodynamic responses that are asymptomatic at rest.

### 4.1 Response to Individual Sighs

As expected (Vaschillo et al. 2015; Vaschillo and Vaschillo 2020), at the level of the individual volitional sigh (Figure 2), peak inhalation aligned with immediate HR acceleration. This response was as expected from the elevation in HR observed during naturalistic sighs (Ramirez 2014) and the phasic relationship between HR and respiration (Yasuma and Hayano 2004). The vascular responses to a sigh followed a time course that suggests that, during inhalation, pulmonary oxygen levels increase and central vessels dilate to accommodate increased blood volume for optimized oxygen transport. This vasodilation results in a dip in central blood pressure, which then signals a centrally mediated sympathetic response to constrict vessels for faster blood flow, elevating MAP. Within a few beats after peak inspiration, characteristic sympathetic signals, i.e., higher MAP and shorter PTT (i.e., vasoconstriction), were observed. Post‐sigh recovery to baseline levels seemed to be passive, exhibiting inter‐individual and inter‐sigh variability. This response timeline to a deliberate sigh provides important mechanistic insight into how repetitive volitional sighing can be used to generate a graded allostatic load (i.e., act as stress test): the sigh sets off a physiological chain reaction that takes several beats to peak and several more beats to dissipate.

Across repeated volitional sighs, neither the amplitude of change nor subsequent recovery timeline notably changed. While individual differences in cardiovascular functioning make group‐averaging of time series data difficult to depict, a representative illustration of this response (Figure 2) shows the modal response pattern across the sample. Figure 3 further illustrates this as a consistent spectral signature of peaks at the sighing frequency with harmonics as expected from the nature of the vasculature and blood flow. The long‐interval sighing task yielded spectral peaks at multiples of 0.033 Hz (e.g., 0.066, 0.099 Hz). On average, the short‐interval sighing task produced peaks multiples of 0.066 Hz as well as a peak close to 0.033 Hz. This replicates a prior study wherein this same pattern of spectral peaks was observed even when the sighing tasks were counterbalanced (Vaschillo and Vaschillo 2020). Based on RSA, the presence of a corresponding cardiac oscillation during the sighing tasks is not surprising, but the magnitude of the power peak may reflect that “forcing” the heart into this oscillatory pattern is effortful. Whether the power peak at the sigh frequency is a useful predictor of individual differences in stress responding warrants further assessment (Vaschillo et al. 2018). Oscillations at 0.066 Hz in the HRV spectrum have been proposed as a measure of arterial elasticity (Vaschillo and Vaschillo 2020).

### 4.2 FIVS as a Graded Stress Protocol

The FIVS protocol used repetitive, deliberately initiated sigh‐like breathing to evoke a progressive system load much in the same way at an exercise‐based stress test is performed in a cardiology office. Assessing function when a physiological system is taxed can be used clinically to reveal subtle impairments that are not evident at rest (Chantler and Frisbee 2020; Santos‐Ribeiro et al. 2018). Research can use similar approaches to identify changes in stress reactivity, and mechanisms underlying these changes, in healthy individuals well prior to disease onset. For example, there is evidence that young binge drinkers show altered cardiovascular reactivity and blunted recovery from stress (Vaschillo et al. 2008; Piano 2017); yet, young adults are typically robust against diagnosable pathophysiological changes even when they engage in a broad array of unhealthy lifestyle behaviors, like binge drinking, insufficient sleep, poor diet, sedentary behavior, and high stress. The allostatic theory suggests that disease is an endpoint of a slow, insidious accumulation of subtle dysfunction that arises from chronic or frequent stress exposure (Sterling 2012; McEwen 1998). The FIVS protocol could be used to better characterize when dysfunction arises.

This study focused on deconstructing the response patterns of individual cardiovascular control processes to the FIVS protocol to more precisely determine the participation of the sympathetic and parasympathetic nervous systems. Using mixed models with a priori cardiorespiratory control variables, we observed that volitional sighing directly affected cardiac activity and engaged the vasculature. In general, as the protocol progressed, cardiac and vascular responses grew. HR, LF‐HRV, PTTv, and LF‐BPV changed from baseline to long‐interval sighing and from long‐ to short‐ interval sighing. Whether the larger responses to the more intense, short‐interval sighing task was due to the faster sighing pace or time under load is unclear because the tasks were not counterbalanced, but a prior study that counterbalanced three tasks with varying sighing frequencies (1 per 15, 30, or 50 s) showed robust cardiovascular and respiratory effects that aligned with sighing intensity (Vaschillo et al. 2018). That study also suggested that young adults who reported frequent binge drinking compared to those who reported lower levels of drinking had evidence of incomplete recovery between sighs when they were paced every 15 s; this was proposed to demonstrate that sighing every 15 s was a sufficient cardiovascular challenge to unveil deterioration in ostensibly healthy young people who routinely engaged in unhealthy lifestyle behaviors.

HR increased during the FIVS protocol in relation to sigh frequency. Respiration plays a large role in rapidly changing HR; yet, these models controlled for the effects of RSA; thus, changes in the current study can be attributed to autonomic, not respiratory control processes. One possible explanation is that sympathetic excitation occurred through increased atrial filling to trigger the Bainbridge reflex that increases HR to improve blood flow out of the heart. However, faster HR could be driven by sympathetic activation and/or vagal withdrawal. Thus, we independently characterized sighing‐induces changes in LF‐HRV and HF‐HRV, both controlling for each other and RSA.

LF‐HRV, a complex marker that has been associated with baroreflex functioning, sympathetic tone, and vagal activity, depending on the respiratory conditions (Shaffer et al. 2014), showed progressive increases across the FIVS protocol. The transition of breathing from an involuntary to voluntarily controlled process likely generates a cognitive load as does maintaining the timing of each sigh; this may be reflected in the LF‐HRV increases. HF‐HRV, an index of parasympathetic activity was not significantly changed during the less intense, long‐interval sighing task; significant decreases below baseline were observed only during the more intense, short‐interval sighing task. Thus, parasympathetically mediated vagal withdrawal may be a secondary effect of sigh‐induced stress loading, only evident as the burden of the task increases. Interestingly, the slower (30 s) but not faster (15 s) sighing pace elicited a power peak ~0.1 Hz (Figure 3), which may indicate indirect baroreflex activation (Vaschillo et al. 2010) and neural engagement.

Using statistical models to isolate vascular responding to the FIVS tasks, we observed an increase in average PTTv suggesting sympathetic activation. PTTv was significantly higher during the short‐ versus long‐interval sighing task. Increased tidal volume, such as a deep inhalation during a volitional sigh, is known to elicit central vasodilation, which, in a healthy system, is followed by vasoconstriction to speed up tissue blood delivery and curb vascular resistance (Chaudhry et al. 2025). This vasomotor variability should increase during the sighing tasks as a direct response to respiratory‐driven blood flow demand, but it also may be dependent on a person's arterial elasticity—the vessels' ability to respond to autonomic control (Vaschillo and Vaschillo 2020). The increased variance during short‐interval sighing likely reflects individual differences in executing rapid sigh‐like behaviors and restoring vascular homeostasis between sighs as time under load and stress burden increase. Such differences in vascular dynamics support the strength of the FIVS protocol as a brief cardiovascular challenge.

Mean blood pressure can also provide insight into the functionality of the cardiovascular system and neural control processes. The primary (albeit not only) role of the baroreflex is to stabilize blood pressure and does so by altering HR and stroke volume at the level of the heart and vessel diameter (Vaschillo et al. 2012). The FIVS protocol led to increases in MAP during the more challenging short‐interval, but not long‐interval sighing task. Since these analyses controlled for the influence of HR and vascular tone, it is possible that this increased pressure is attributable to changes in stroke volume. In young, healthy individuals, changes in stroke volume during stress are typically modest due to the resilient nature of the myocardium; individual differences in stroke volume tend to be more attributable to athleticism and fitness than to disease. Yet, stroke volume is an important metric of cardiovascular disease risk due to its drastic effect on blood flow; low stroke volume is a hallmark feature of heart failure. Thus, a failure to respond to fast sighing with increased MAP may reflect a loss of dynamic responding in stroke volume. However, because this study used statistical modeling of MAP to proxy stroke volume, future studies are needed to directly measure stroke volume during sighing or similar sympathetic challenge.

Since blood pressure stabilization is typically paramount, rhythmically oscillations that return blood pressure to homeostasis after perturbation are commonly observed. The significance of these oscillations remains unclear but LF‐BPV likely reflects vasomotor tone and vascular resistance (Parati et al. 1995), whereas HF‐BPV appears more directly driven by the mechanical effects of respiration, such as from tidal volume. Notably, LF‐BPV showed a graded response to sighing frequency, with significant increases from baseline to slow sighing and from slow sighing to fast sighing. On the other hand, HF‐BPV responses were comparable between frequencies, which may indicate that mechanical effort to sigh is consistent across the protocol and that the effects dissipate after each sigh, even when sighing frequency is doubled.

### 4.3 Sex Differences

In all statistical models, substantial individual variability was noted. These individual differences may be related to differences in physiological health or health behavior profiles that warrant further examination. In fact, the data for this study comes from the laboratory component of an ongoing 2‐year behavioral data collection study that seeks to link early changes in stress reactivity to substance use, sleep behaviors, physical activity, and perceived stress. While future analyses will delve into these behavioral data, we sought to first explore differential cardiovascular responses to the sighing tasks between men and women as a preliminary step toward dissecting the factors that impact sighing reactivity. This was based on evidence that men and women physiologically respond to stress differently (Charkoudian and Rabbitts 2009). As expected from substantive prior literature, baseline differences were observed. Males had lower heart rates and higher LF‐HRV (Koenig and Thayer 2016; Lutfi and Sukkar 2011) than females. Males also had higher PTTv; however, the interpretation of this finding is unclear due to the large variance in responses to the short‐interval sighing task in men.

Response to sighing also demonstrated a distinct pattern of sex differences. Females consistently demonstrated attenuated responses to sighing compared to males, except in the case of HF‐HRV, the only vagal index, which was more responsive to sighing. Recent literature has suggested that there are cardioprotective properties in female cardiovascular tone, resulting in moderated responses to sympathetic stimuli (Charkoudian and Rabbitts 2009; Dart et al. 2002). This raises the possibility that male cardiovascular systems are more volatile under stress than those of females but this may be offset by lower resting HR and higher vagal tone. Moreover, given that no sex differences in blood pressure were observed, it may be that male and female cardiovascular systems simply rely on different cardiovascular mobilization pathways to support stable blood pressure under stress, at least when they are young and healthy. This is further supported by the smaller magnitude of changes in blood pressure variability in females. Together, these observations demonstrate that the FIVS protocol taxes the system sufficiently to reveal sex‐specific differences in cardiovascular regulation. The findings, more generally, point to a need for sex‐specific strategies for early identification and prevention of stress‐related cardiovascular dysfunction. Further, these differences in sympathetic responsivity emphasize the need for more involved sex‐differentiated psychophysiological studies.

### 4.4 Limitations

Participants in this study were college students. Health behaviors (e.g., sleep, exercise, and substance use), which can influence autonomic activity, were not considered in these analyses but may partially explain individual differences that were observed. Future analyses will delve deeper into how lifestyle behaviors can change these effects. Likewise, while individuals with existing health conditions that directly affect cardiovascular functioning, such as arrhythmias, were excluded, this study was otherwise liberal in its inclusion criteria. Health conditions that indirectly affect cardiovascular functioning, or those that have not been formally diagnosed, could also affect stress reactivity. Further, while those who could be clinically classified as underweight or obese were excluded from the study, anthropometric factors such as BMI could also contribute to interindividual variability in the current study. While this study primarily focused on ostensibly healthy subjects, it is important to acknowledge the variety of mental and physical health factors that could affect individual stress reactivity and thus, response to the FIVS protocol.

The observed changes in HR and blood pressure were small and not necessarily clinically relevant, but it is important to note that subtle changes in young, robust cardiovascular systems may nonetheless be meaningful indicators of long‐term health trajectories. These cardiovascular indices were examined over the full task periods (5 min), which aligns with current analytical guidelines for some measures (e.g., HRV). However, more complex time‐varying analyses could enable evaluation of inter‐sigh variability and beat‐by‐beat physiological fluctuations. We also do not know whether this volitional sighing task is directly affecting the autonomic nervous system as a naturalistic sigh does. It could be that more skeletal muscle is activated during the volitional sigh than during normal respiration, necessitating these robust cardiovascular changes to the quick and large increase in blood demand.

We deemed a simplified peak‐to‐peak estimation of RSA sufficient to control for respiration‐driven cardiac effects in these task‐wide analyses—more complex algorithms that follow the change in HR throughout each respiration do exist (Lewis et al. 2012) and may be warranted in future studies that examine beat‐to‐beat changes during sighing. Lastly, we did not measure “sigh performance,” i.e., a threshold for which a volitional sigh would generate a sufficient load to match a naturalistic sigh. It is possible that sigh amplitude, respiratory period, and breathing technique all play a role in sighing. Future analyses that more precisely evaluate respiratory physiology are needed. Nonetheless, the present study reports autonomic responses that were robust enough to reach significance despite the relatively low proportion of volitional sighs to normal breaths.

## 5 Conclusion

During rest states, one naturalistic sigh occurs every ~5 min in adults. This interval may be adequate for internal regulation of static metabolic state, with the single sigh serving as a brief sympathetic stimulus on the backdrop of high parasympathetic tone to ensure cardiac and vascular function remain near homeostatic set points. During acute stress states, the rate of naturalistic sighing increases; this may serve to sustain the elevated blood flow needs for both greater metabolism and central attentional processing. This study further suggests that repetitive, volitional sighing at fixed intervals can be used as a progressive sympathetic challenge (i.e., stress test). There was a clear pattern of sympathetic responses to the FIVS protocol with some evidence of secondary process involvement, namely vagal withdrawal and stroke volume, over time under load as difficulty increased. While more research is needed, this study provides initial evidence that suggests the FIVS protocol can be used to progressively load the cardiovascular system beyond metabolic and cognitive needs to reveal subtle changes in stress responses.

## Author Contributions

**Neel Muzumdar:** formal analysis, data curation, writing – original draft, writing – review and editing, visualization, investigation. **Kelly Sun:** data curation, investigation, methodology, project administration. **Samuel Zhang:** software, visualization, data curation. **Kelsey Piersol:** supervision, investigation, methodology, data curation. **Anthony P. Pawlak:** supervision, formal analysis, software. **Marsha E. Bates:** supervision, resources. **Jennifer F. Buckman:** validation, methodology, conceptualization, investigation, funding acquisition, writing – review and editing, supervision, resources, project administration.

## Funding

This work was supported by the National Institute of Alcohol Abuse and Alcoholism (R01AA027017, J.F.B.; F31AA031620, N.M.).

## Conflicts of Interest

The authors declare no conflicts of interest.

## Supporting information


**Data S1:** psyp70235‐sup‐0001‐Supinfo1.docx.

## Data Availability

The data that support the findings of this study are available from the corresponding author upon reasonable request.
