# Supplementary material for: Dissecting Cardiovascular Responses to a Fixed‐Interval Volitional Sighing Protocol Using a Mixed Modeling Approach
Source: Psychophysiology. 2026 Jan 16;63(1):e70235. doi: 10.1111/psyp.70235 (PMC12811738; doi:10.1111/psyp.70235)
Supplement: Supplementary file 1 — Data S1: psyp70235‐sup‐0001‐Supinfo1.docx. [file PSYP-63-e70235-s001.docx]

**SUPPLEMENTAL MATERIALS**

***LMM Specifications & Diagnostics.*** All LMMs were fitted using restricted maximum likelihood estimation (REML) and robust standard errors were specified because they are less sensitive to departures from normality. Partial models for each LMM, with either the effects of interest or the *a priori* cardiorespiratory covariates, were created, followed by the final model including all effects. All models included a random intercept of subjects to account for interindividual variability. Influential data points in the partial models were assessed at the individual task value level using Cook’s *D* and restricted likelihood distance (RLD), wherein observations with values >=1 were excluded. Influential data points in the final model were assessed at the individual task value level using studentized residuals, Cook’s *D*, the PRESS statistic, and RLD. Dot and needle plots were constructed for Cook’s *D* and the RLD, and an index plot for the PRESS values, which were used for visual inspection of values that stood out from the main distribution of each statistic. Observations associated with studentized residuals >= |4|, Cook’s *D* or RLD values >= 1, or those that stood out from the main distribution of their respective plots were flagged as influential data points and removed from the analyses. All data point removals improved the Q-Q plot overall.

Influence diagnostic statistics for the final LMM of HR regressed onto task and sex, with PWV and RSA as covariates, showed that there were 2 observations that were influential as visual outliers in the RLD needle plot, which were removed from the subsequent analysis. The LMM was rerun (N_person_=230 with 687 total observations; 61 daily observations were not accepted by the model because of missing data issues).

Influence diagnostic statistics for the final LMM of LF-HRV regressed onto task and sex, with RSA and HF-HRV as covariates, showed that there was 1 observation that was influential with studentized residuals >= |4|, which was removed from the subsequent analysis. The LMM was rerun (N_person_=236 with 707 total observations; 42 daily observations were not accepted by the model because of missing data issues).

Influence diagnostic statistics for the final LMM of HF-HRV regressed onto task and sex, with RSA and LF-HRV as covariates, showed that there was a total of 3 observations that were influential, 1 that was a visual outlier in the RLD needle plot, and 2 with studentized residuals >= |3|, which were removed from the subsequent analysis. The LMM was rerun (N_person_=236 with 705 total observations; 42 daily observations were not accepted by the model because of missing data issues).

Influence diagnostic statistics for partial LMM of PTTv regressed onto task and sex, showed that there was a total of 17 observations that were influential with RLD >=1, which were removed from the subsequent analysis. The LMM was rerun (N_person_=246 with 726 total observations; 6 observations were not accepted by the model because of missing data issues). Statistics for the partial LMM of PTTv regressed onto heart rate showed that there was a total of 15 observations that were influential with RLD >=1, which were removed from the subsequent analysis. The LMM was rerun (N_person_=247 with 729 total observations; 6 observations were not accepted by the model because of missing data issues). Statistics for the final LMM of PTTv regressed onto task and sex, with heart rate as a covariate, showed that there was a total of 38 observations that were influential, 1 with Cook’s *D* >= 1 and RLD >= 1, 15 with RLD => 1, and 22 that were visual outliers in the PRESS index plot, which were removed from the subsequent analysis. The LMM was rerun (N_person_=246 with 706 total observations; 6 observations were not accepted by the model because of missing data issues).

Influence diagnostic statistics for the final LMM of MAP regressed onto task and sex, with HR and PWV as covariates, showed that there was a total of 9 observations that were influential, 3 that were visual outliers in the PRESS index plot, 4 that were visual outliers in the scatterplot, and 2 with studentized residuals >= |4|, which were removed from the subsequent analysis. The LMM was rerun (N_person_=241 with 716 total observations; 24 daily observations were not accepted by the model because of missing data issues).

Influence diagnostic statistics for both partial LMMs showed the same 1 observation that was influential with RLD >=1, which was removed from the subsequent analyses. The LMM of LF-BPV regressed onto task and sex was rerun (N_person_=250 with 745 total observations; 4 observations were not accepted by the model because of missing data issues). The LMM of LF-BPV regressed onto RSA and HF-BPV was rerun (N_person_=236 with 705 total observations; 44 observations were not accepted by the model because of missing data issues). Statistics for the final LMM of LF-BPV regressed onto task and sex, with RSA and HF-BPV as covariates, showed that there was a total of 8 observations that were influential, 1 with RLD => 1, 2 that were visual outliers in the PRESS index plot, and 6 that were visual outliers in the residual scatterplot, which were removed from the subsequent analysis. The LMM was rerun (N_person_=236 with 702 total observations; 41 observations were not accepted by the model because of missing data issues).

Influence diagnostic statistics for the final LMM of HF-BPV regressed onto task and sex, with RSA and LF-BPV as covariates, showed that there was 1 observation that was influential, with an RLD >=1, which were removed from the subsequent analysis. The LMM was rerun (N_person_=236 with 705 total observations; 42 daily observations were not accepted by the model because of missing data issues).

**Table 1**. Null Linear Mixed Model Results for Cardiovascular Variables

| **Variable** | **Intercept (A)** | **t** | **df** | **Variance (σ²)** | **Z** | **ICC** |
| --- | --- | --- | --- | --- | --- | --- |
| HR | 75.337 | 116.59* | 249 | 102.48 | 10.91* | 0.936 |
| LF-HRV | 9.405 | 116.59* | 249 | 0.441 | 8.86* | 0.575 |
| HF-HRV | 8.158 | 126.18* | 249 | 0.992 | 10.54* | 0.853 |
| PTTv | 58.472 | 10.52* | 248 | 4841.74 | 6.75* | 0.36 |
| MAP | 81.044 | 86.85* | 249 | 215.82 | 11.02* | 0.964 |
| LF-BPV | 4.943 | 100.91* | 249 | 0.453 | 8.27* | 0.505 |
| HF-BPV | 3.001 | 71.63* | 249 | 0.355 | 8.91* | 0.582 |
| *Note.* HR = heart rate; LF-HRV = low-frequency heart rate variability; HF-HRV = high-frequency heart rate variability; PTTv = pulse transit time variability; MAP = mean arterial pressure; LF-BPV = low-frequency blood pressure variability; HF-BPV = high-frequency blood pressure variability. ICC = intraclass correlation coefficient. Significant results (p<0.05) are denoted by *. | | | | | | |

Table 2. Hierarchical Linear Mixed Model Results for Heart Rate

| **Model** | **DF1, DF2** | **F** | **AIC** | **BIC** |
| --- | --- | --- | --- | --- |
| **Model A** |  |  | **4386.2** | **4393.2** |
| Task | 2, 494 | 56.19* |  |  |
| Sex | 1, 494 | 17.22* |  |  |
| Task x Sex | 2, 494 | 9.89* |  |  |
| **Model B** |  |  | **4103.2** | **4110.1** |
| PWV | 1, 457 | 27.98* |  |  |
| RSA | 1, 457 | 8.75 |  |  |
| **Final Model (A+B)** |  |  | **3931.2** | **3938** |
| Task | 2, 451 | 54.15* |  |  |
| Sex | 1, 451 | 20.46* |  |  |
| Task x Sex | 2, 451 | 10.02* |  |  |
| PWV | 1, 451 | 28.56* |  |  |
| RSA | 1, 451 | 4.76* |  |  |
| *Note*. Results shown are Type III tests of fixed effects. The hierarchical sequence was as follows: Model A included the primary predictors: task, sex, and the task × sex interaction. Model B tested only the physiological covariates: pulse wave velocity (PWV) and respiratory sinus arrhythmia (RSA). The final model combined both sets of predictors into a single model. Significant results (p<0.05) are denoted by *. The random-effects structure was held constant across all steps. Model fit statistics (AIC, BIC) are reported for each step. Lower AIC/BIC indicates better model fit. | | | | |

Table 3. Hierarchical Linear Mixed Model Results for Low-Frequency Heart Rate Variability

| **Model** | **DF1, DF2** | **F** | **AIC** | **BIC** |
| --- | --- | --- | --- | --- |
| **Model A** |  |  | **1405.8** | **1412.9** |
| Task | 2, 494 | 110.79* |  |  |
| Sex | 1, 494 | 26.26* |  |  |
| Task x Sex | 2, 494 | 0.28 |  |  |
| **Model B** |  |  | **1234.4** | **1241.3** |
| RSA | 1, 470 | 124.91* |  |  |
| HF-HRV | 1, 470 | 517.17* |  |  |
| **Final Model (A+B)** |  |  | **930.1** | **937.1** |
| Task | 2, 465 | 119.70* |  |  |
| Sex | 1, 465 | 48.18* |  |  |
| Task x Sex | 2, 465 | 2.05 |  |  |
| RSA | 1, 465 | 69.85* |  |  |
| HF-HRV | 1, 465 | 583.53* |  |  |
| *Note*. Results shown are Type III tests of fixed effects. The hierarchical sequence was as follows: Model A included the primary predictors: task, sex, and the task × sex interaction. Model B tested only the physiological covariates: respiratory sinus arrhythmia (RSA) and high-frequency heart rate variability (HF-HRV). The final model combined both sets of predictors into a single model. Significant results (p<0.05) are denoted by *. The random-effects structure was held constant across all steps. Model fit statistics (AIC, BIC) are reported for each step. Lower AIC/BIC indicates better model fit. | | | | |

Table 4. Hierarchical Linear Mixed Model Results for High-Frequency Heart Rate Variability

| **Model** | **DF1, DF2** | **F** | **AIC** | **BIC** |
| --- | --- | --- | --- | --- |
| **Model A** |  |  | **1517.4** | **1524.4** |
| Task | 2, 494 | 27.75* |  |  |
| Sex | 1, 494 | 1.07 |  |  |
| Task x Sex | 2, 494 | 0.95 |  |  |
| **Model B** |  |  | **1322.1** | **1329.1** |
| RSA | 1, 470 | 17.03* |  |  |
| LF-HRV | 1, 470 | 153.76* |  |  |
| **Final Model (A+B)** |  |  | **1169** | **1176** |
| Task | 2, 463 | 71.53* |  |  |
| Sex | 1, 463 | 3.14 |  |  |
| Task x Sex | 2, 463 | 3.44* |  |  |
| RSA | 1, 463 | 14.65* |  |  |
| LF-HRV | 1, 463 | 273.51* |  |  |
| *Note*. Results shown are Type III tests of fixed effects. The hierarchical sequence was as follows: Model A included the primary predictors: task, sex, and the task × sex interaction. Model B tested only the physiological covariates: respiratory sinus arrhythmia (RSA) and low-frequency heart rate variability (LF-HRV). The final model combined both sets of predictors into a single model. Significant results (p<0.05) are denoted by *. The random-effects structure was held constant across all steps. Model fit statistics (AIC, BIC) are reported for each step. Lower AIC/BIC indicates better model fit. | | | | |

Table 5. Hierarchical Linear Mixed Model Results for Pulse Transit Time Variability

| **Model** | **DF1, DF2** | **F** | **AIC** | **BIC** |
| --- | --- | --- | --- | --- |
| **Model A** |  |  | **6677.3** | **6684.3** |
| Task | 2, 477 | 78.76* |  |  |
| Sex | 1, 477 | 16.28* |  |  |
| Task x Sex | 2, 477 | 8.58* |  |  |
| **Model B** |  |  | **7034.5** | **7041.5** |
| HR | 1, 482 | 0.18 |  |  |
| **Final Model (A+B)** |  |  | **6099** | **6106** |
| Task | 2, 456 | 98.96* |  |  |
| Sex | 1, 456 | 15.40* |  |  |
| Task x Sex | 2, 456 | 9.23* |  |  |
| HR | 1, 456 | 1.46 |  |  |
| *Note*. Results shown are Type III tests of fixed effects. The hierarchical sequence was as follows: Model A included the primary predictors: task, sex, and the task × sex interaction. Model B tested only the physiological covariates: heart rate (HR). The final model combined both sets of predictors into a single model. Significant results (p<0.05) are denoted by *. The random-effects structure was held constant across all steps. Model fit statistics (AIC, BIC) are reported for each step. Lower AIC/BIC indicates better model fit. | | | | |

Table 6. Hierarchical Linear Mixed Model Results for Mean Arterial Pressure

| **Model** | **DF1, DF2** | **F** | **AIC** | **BIC** |
| --- | --- | --- | --- | --- |
| **Model A** |  |  | **4619.9** | **4626.9** |
| Task | 2, 487 | 18.17* |  |  |
| Sex | 1, 487 | 0.42 |  |  |
| Task x Sex | 2, 487 | 1.14 |  |  |
| **Model B** |  |  | **4602.5** | **4609.5** |
| HR | 1, 481 | 24.18* |  |  |
| PWV | 1, 481 | 2.36 |  |  |
| **Final Model (A+B)** |  |  | **4439.9** | **4446.8** |
| Task | 2, 469 | 6.66* |  |  |
| Sex | 1, 469 | 0.50 |  |  |
| Task x Sex | 2, 469 | 0.91 |  |  |
| HR | 1, 469 | 11.12* |  |  |
| PWV | 1, 469 | 8.02* |  |  |
| *Note*. Results shown are Type III tests of fixed effects. The hierarchical sequence was as follows: Model A included the primary predictors: task, sex, and the task × sex interaction. Model B tested only the physiological covariates: heart rate (HR) and pulse wave velocity (PWV). The final model combined both sets of predictors into a single model. Significant results (p<0.05) are denoted by *. The random-effects structure was held constant across all steps. Model fit statistics (AIC, BIC) are reported for each step. Lower AIC/BIC indicates better model fit. | | | | |

Table 7. Hierarchical Linear Mixed Model Results for LF Blood Pressure Variability

| **Model** | **DF1, DF2** | **F** | **AIC** | **BIC** |
| --- | --- | --- | --- | --- |
| **Model A** |  |  | **1375** | **1382.1** |
| Task | 2, 491 | 218.17* |  |  |
| Sex | 1, 491 | 73.35* |  |  |
| Task x Sex | 2, 491 | 7.05* |  |  |
| **Model B** |  |  | **1234.4** | **1241.3** |
| RSA | 1, 470 | 124.91* |  |  |
| HF-BPV | 1, 470 | 517.17* |  |  |
| **Final Model (A+B)** |  |  | **1145.2** | **1152.1** |
| Task | 2, 460 | 112.12* |  |  |
| Sex | 1, 460 | 68.82* |  |  |
| Task x Sex | 2, 460 | 3.13* |  |  |
| RSA | 1, 460 | 8.68* |  |  |
| HF-BPV | 1, 460 | 49.24* |  |  |
| *Note*. Results shown are Type III tests of fixed effects. The hierarchical sequence was as follows: Model A included the primary predictors: task, sex, and the task × sex interaction. Model B tested only the physiological covariates: respiratory sinus arrhythmia (RSA) and high-frequency blood pressure variability (HF-BPV). The final model combined both sets of predictors into a single model. Significant results (p<0.05) are denoted by *. The random-effects structure was held constant across all steps. Model fit statistics (AIC, BIC) are reported for each step. Lower AIC/BIC indicates better model fit. | | | | |

Table 8. Hierarchical Linear Mixed Model Results for HF Blood Pressure Variability

| **Model** | **DF1, DF2** | **F** | **AIC** | **BIC** |
| --- | --- | --- | --- | --- |
| **Model A** |  |  | **1220.3** | **1227.3** |
| Task | 2, 492 | 134.06* |  |  |
| Sex | 1, 492 | 20.16* |  |  |
| Task x Sex | 2, 492 | 8.95* |  |  |
| **Model B** |  |  | **1322.1** | **1329.1** |
| RSA | 1, 467 | 4.55* |  |  |
| LF-BPV | 1, 467 | 294.19* |  |  |
| **Final Model (A+B)** |  |  | **1044.3** | **1051.2** |
| Task | 2, 463 | 27.94* |  |  |
| Sex | 1, 463 | 5.60* |  |  |
| Task x Sex | 2, 463 | 4.28* |  |  |
| RSA | 1, 463 | 1.01 |  |  |
| LF-HRV | 1, 463 | 57.07* |  |  |
| *Note*. Results shown are Type III tests of fixed effects. The hierarchical sequence was as follows: Model A included the primary predictors: task, sex, and the task × sex interaction. Model B tested only the physiological covariates: respiratory sinus arrhythmia (RSA) and low-frequency blood pressure variability (LF-BPV). The final model combined both sets of predictors into a single model. Significant results (p<0.05) are denoted by *. The random-effects structure was held constant across all steps. Model fit statistics (AIC, BIC) are reported for each step. Lower AIC/BIC indicates better model fit. | | | | |
